# 2110. Immunocompromised Adults Hospitalized with Community-Acquired Pneumonia in the United States: Incidence and Clinical Outcomes

**DOI:** 10.1093/ofid/ofac492.1731

**Published:** 2022-12-15

**Authors:** Julio A Ramirez, Thomas R Chandler, Stephen Furmanek, Ruth Carrico, Ashley M Wilde, Daniya Sheikh, Raghava Sekhar Ambadapoodi, Vidyulata Salunkhe, Mohommad T Tahboub, Forest W Arnold, Jose Bordon, Rodrigo Cavallazzi

**Affiliations:** Norton Healthcare, Louisville, Kentucky; Norton Healthcare, Louisville, Kentucky; Norton Healthcare, Louisville, Kentucky; Norton Healthcare, Louisville, Kentucky; Norton Healthcare, Louisville, Kentucky; University of Louisville School of Medicine, Louisville, Kentucky; University of Louisville, Louisville, Kentucky; University of Louisville School of Medicine, Louisville, Kentucky; University of Louisville School of Medicine, Louisville, Kentucky; University of Louisville School of Medicine, Louisville, Kentucky; George Washington University, Washington, District of Columbia; University of Louisville School of Medicine, Louisville, Kentucky

## Abstract

**Background:**

Community-acquired pneumonia (CAP) is an important complication in immunocompromised adults (ICAs). In hospitalized patients, the burden of CAP in ICAs is not well defined. The primary objective of this study was to define incidence, epidemiology, and outcomes of ICAs hospitalized with CAP in the city of Louisville. The secondary objective of this study was to estimate the burden of CAP in ICAs in the US.

**Methods:**

This was a prospective population-based cohort study of consecutive hospitalized adult Louisville residents with CAP at all adult hospitals in Louisville from 1 June 2014 to 31 May 2016. An ICA was defined as a patient with any of the following medical conditions or treatments: (1) primary immunodeficiency disease; (2) advanced-stage cancer (stage III or IV cancer or hematologic cancer); (3) advanced HIV infection (CD4 T-lymphocyte count < 200 cells/mL or < 14%); (4) solid organ transplantation; (5) hematopoietic stem cell transplantation; (6) receiving cancer chemotherapy; (7) receiving biological immune modulators; (8) receiving corticosteroid therapy with a dose ≥ 20 mg prednisone or equivalent daily for at least 14 days prior to hospitalization; or (9) receiving disease-modifying antirheumatic drugs (DMARDs). The annual population-based CAP incidence among ICAs was calculated. Mortality was evaluated during hospitalization and at 30 days, 6 months, and 1 year after hospitalization.

**Results:**

A total of 7449 unique patients were included, with 854 (11%) ICAs identified. **Figure 1** depicts the immunocompromising conditions. Per 100,000 adults in Louisville, each year 75 ICAs are hospitalized with CAP (95% CI: 68-78), corresponding to an estimated 190,106 ICAs hospitalized with CAP in the US. Mortality during hospitalization was 8% in ICAs vs 6% in non-ICAs; at 30 days 23% in ICAs vs 11% in non-ICAs, at 6 months 42% in ICAs vs 20% in non-ICAs, and at 1 year 52% in ICAs vs 27% in non-ICAs.

**Figure 1:**
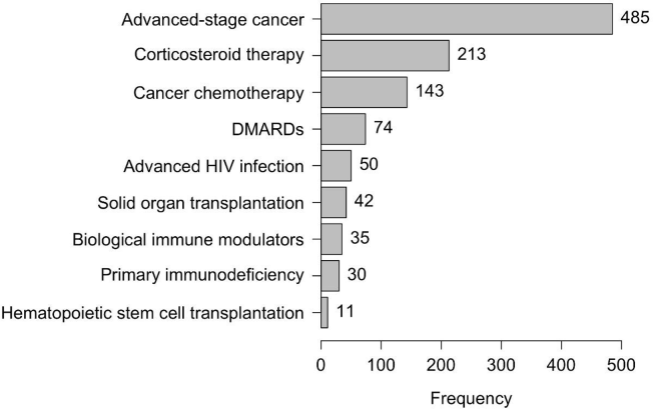
Frequency of Immunocompromising Conditions and Treatments

**Conclusion:**

This study indicates that nearly one out of nine hospitalized CAP patients are immunocompromised, and 190,000 ICAs may be hospitalized due to CAP each year in the US. We found a twofold increase in mortality at every time point after discharge for ICAs compared to non-ICAs hospitalized due to CAP. Further strategies to prevent CAP and improve outcomes in ICAs hospitalized with CAP are necessary.

**Disclosures:**

**Forest W. Arnold, DO, MSc**, Gilead Sciences, Inc.: Grant/Research Support.

**Figure 1:** Frequency of Immunocompromising Conditions and Treatments

Immunocompromising conditions and treatments among 7449 patients hospitalized for community-acquired pneumonia sorted by frequency. Abbreviations: DMARDs: Disease-modifying antirheumatic drugs; HIV: Human immunodeficiency virus.

